# Tumor Treating Fields (TTFields) demonstrate antiviral functions *in vitro*, and safety for application to COVID-19 patients in a pilot clinical study

**DOI:** 10.3389/fmicb.2023.1296558

**Published:** 2023-11-29

**Authors:** Avraham Abutbul, Helena Mumblat, Yaara Porat, Nehemya Friedman, Nofar Atari, Shirley Sharabi, Ahmad Nama, Waseem Mugahed, Asa Kessler, Yotam Kolben, Reuben Ruby Shamir, Doron Manzur, Ori Farber, Liora Bosch, Gitit Lavy-Shahaf, Eyal Dor-On, Adi Haber, Moshe Giladi, Uri Weinberg, Yoram Palti, Yael Mardor, Michal Mandelboim

**Affiliations:** ^1^Medical Intensive Care Unit, Hadassah-Hebrew University Medical Center, Jerusalem, Israel; ^2^Novocure Ltd., Haifa, Israel; ^3^Central Virology Laboratory, Public Health Services, Ministry of Health and Sheba Medical Center, Ramat-Gan, Israel; ^4^The Advanced Technology Center and Radiology Institute, Sheba Medical Center, Ramat-Gan, Israel; ^5^Sackler Faculty of Medicine, Tel-Aviv University, Tel Aviv-Yafo, Israel

**Keywords:** Tumor Treating Fields (TTFields), antiviral activity, clinical safety, coronavirus, COVID-19

## Abstract

Coronaviruses are the causative agents of several recent outbreaks, including the COVID-19 pandemic. One therapeutic approach is blocking viral binding to the host receptor. As binding largely depends on electrostatic interactions, we hypothesized possible inhibition of viral infection through application of electric fields, and tested the effectiveness of Tumor Treating Fields (TTFields), a clinically approved cancer treatment based on delivery of electric fields. In preclinical models, TTFields were found to inhibit coronavirus infection and replication, leading to lower viral secretion and higher cell survival, and to formation of progeny virions with lower infectivity, overall demonstrating antiviral activity. In a pilot clinical study (NCT04953234), TTFields therapy was safe for patients with severe COVID-19, also demonstrating preliminary effectiveness data, that correlated with higher device usage.

## Introduction

Coronaviruses, enveloped single-stranded RNA viruses, cause Severe Acute Respiratory Syndrome (SARS), Middle-East Respiratory Syndrome (MERS), and Coronavirus Disease 2019 (COVID-19) (Haque et al., 2020; Hu et al., 2021; Liu et al., 2021). The spectrum of COVID-19 illness ranges from asymptomatic infection to severe pneumonia with acute respiratory distress syndrome (ARDS) and death. As successful viral infection requires host cell viral entry, therapeutic strategies mostly focus on blocking viral-cell binding (Huang Y. et al., 2020), which largely depends on electrostatic protein–protein interactions (Sheinerman et al., 2000). Specifically, the high receptor binding affinity of SARS Coronavirus 2 (SARS-CoV-2, responsible for COVID-19), has been attributed to the large positive charge of its spike (S) protein involved in host receptor binding (Hassanzadeh et al., 2020; Huang Y. et al., 2020; Ali et al., 2021; Rezaei et al., 2022). This suggests the possible utilization of electric fields to interfere with viral infection.

Tumor Treating Fields (TTFields) therapy is a clinically-approved, potent and safe cancer treatment based on locoregional non-invasive application of low intensity [1–3 V/cm root-mean-square (RMS)], intermediate frequency (100–500 kHz), electric fields (Mun et al., 2018; Carrieri et al., 2020). Within this frequency range, electric fields do not stimulate nerve cells or cause significant tissue heating. However, they can exert bi-directional forces on polarized molecules within cancer cells to disrupt cell division and downstream processes, causing cell death (Gera et al., 2015; Giladi et al., 2015; Rominiyi et al., 2021).

TTFields therapy is currently applied to the thorax of oncological patients for treatment of two pulmonary cancers (Supplementary Figure S1), pleural mesothelioma (approved, based on STELLAR trial) (Ceresoli et al., 2019) and non-small cell lung carcinoma (LUNAR trial, NCT02973789) (Leal et al., 2023). Improved treatment outcomes have been associated with prolonged device usage time (≥18 h/day) (Ballo et al., 2019; Toms et al., 2019). The main treatment-related adverse event (AE) reported to date is low-grade skin irritation beneath the skin-applied treatment arrays (Stupp et al., 2012, 2015, 2017; Mrugala et al., 2014; Ceresoli et al., 2019; Shi et al., 2020), which in most cases resolves with application of topical steroids or intermittent treatment interruptions (Lacouture et al., 2014).

The current study examined the *in vitro* effects of TTFields on coronavirus infection and evaluated the safety of TTFields in COVID-19 patients.

## Materials and methods

### Cells and virus

Human MRC-5 lung fibroblast cells (ATCC, CCL-171™) were grown in 5% CO_2_ humidified incubator at 37°C in Eagle’s Minimum Essential Medium (EMEM) (ATCC, 30–2003™) supplemented with 10% fetal bovine serum (FBS) (Biological Industries, 04–007-1A). HCoV-229E (ATCC, VR740™) was handled in Biosafety level 2 (BSL2) facilities and grown at its optimal temperature of 35°C. For production of a stock virus pool, the commercial HCoV-229E was grown in MRC-5 cells according to the supplier instructions, quantified by plaque assay, and stored in aliquots at −80°C.

### TTFields application

TTFields (1.5 V/cm RMS) were applied using the inovitro™ system (Novocure, Israel). Cell suspensions were grown in inovitro dishes composed of high dielectric constant ceramic [lead magnesium niobate–lead titanate (PMN-PT)], with two perpendicularly printed pairs of transducers on their outer walls. The transducers were connected to a sinusoidal waveform generator that produces electric fields at selected frequency and intensity, while changing field orientation every 1 s.

### Effect of TTFields on viral entry

MRC-5 cells were seeded on glass cover slips (22 mm diameter 1.5 × 10^5^ cells/coverslip). After 24 h, the cells were transferred into inovitro dishes containing 2 mL EMEM supplemented with 2% FBS. The cells were then exposed to TTFields at 100, 150 or 400 kHz for 30 min and infected with HCoV-229E at multiplicity of infection (MOI) of 0.01 with continued TTFields application. Control cells were not treated with TTFields at any time. At 0.5 or 2 h post infection (hpi) the cells were washed 3 times with PBS, trypsinized, resuspended and counted using a Scepter™ 2.0 Cell Counter (Merck, Millipore). The cells were further analyzed by real-time quantitative reverse transcription PCR (RT-qPCR), as described below.

### Effect of TTFields on long term viral exposure

MRC-5 cells were exposed to 150 kHz TTFields, and 30 min later infected with HCoV-229E, MOI 0.0001. At 3 hpi the cells were washed (3 times with PBS) to remove unbound virus and maintained in fresh media for a total of 24, 48 or 72 h, with continuous TTFields exposure. At treatment end, growth medium was collected for RT-qPCR analysis and plaque assay, and cells were washed 3 times with PBS, trypsinized and analyzed by cell counting and RT-qPCR as described above.

### Effect of TTFields on viral replication

MRC-5 cells were infected with HCoV-229E, MOI 0.01. The cells were washed (3 times with PBS) at 3 hpi, fresh medium was added, and only then 150 kHz TTFields application was initiated for up to 24 hpi, followed by analysis of double strand RNA (dsRNA) formation as described below.

### The effect of TTFields together with remdesivir

MRC-5 cells were exposed to 150 kHz TTFields, and 30 min later infected with HCoV-229E, MOI 0.01. At 3 hpi the cells were washed (3 times with PBS) and fresh media containing 0, 0.011, or 0.023 μM remdesivir (Cayman Chemicals, Cay30354) was added. At 48 hpi, growth media and cells were collected and analyzed by RT-qPCR, or fixed and examined for dsRNA.

### Scanning electron microscopy

MRC-5 cells were seeded on glass coverslips (13 mm diameter, 4 × 10^4^ cells/cover slip), and handled as described above. The cells were exposed to 150 kHz TTFields, and 30 min later infected with HCoV-229E, MOI 20. At 0.5 hpi the slides were transferred to clean plates, gently washed with PBS, and fixed using 2% glutaraldehyde and 1% paraformaldehyde in 0.1 M sodium cacodylate buffer for 2 h at room temperature. Following 15 min fixation with osmium tetroxide in cacodylate buffer, the samples were dehydrated in graded ethanol series, critical point dried (Quorum K850) and sputter coated with 4 nm of iridium (Quorum Q150T). Samples were then viewed on Zeiss Ultra Plus HR Scanning Electron Microscope.

### Transmission electron microscopy

MRC-5 cells were seeded on thermanox coverslips (22 mm diameter (Thermo, 174,977), 3 × 10^4^ cells/coverslip), and handled as described above. The cells were infected with HCoV-229E, MOI 0.03, washed (3 times with PBS) at 3 hpi, and only then exposed to 150 kHz TTFields for up to 48 hpi. The cells were then fixed for 2 h with 2% glutaraldehyde, 3% paraformaldehyde, in 0.1 M sodium cacodylate buffer containing 5 mM CaCl2. The samples were washed, post fixed using 2% osmium tetroxide, washed with DDW, and incubated in 2% uranyl acetate. Following dehydration in graded ethanol series, the coverslips with the cells were moved to fresh wells filled with Epon812 for embedding. 75 nm transverse sections were cut using ultramicrotome UC7 (Leica), transferred to copper grids and viewed using Talos L120C Transmission Electron Microscope at accelerating voltage of 120 keV.

### Real-time quantitative reverse transcription PCR

Total RNA was extracted using the MagnaPure 96 instrument (Roche, Germany) according to the manufacturer instructions. Reactions were performed in 25 μL reaction mixture, prepared with AgPath-ID™ One-Step RT-PCR Reagents (Applied Biosystems, Thermo Fisher Scientific) using type-specific primers and probes (Hylabs, Israel) for HCoV-229E selected with Primer Express software (PE Applied Biosystems) based on the genomic regions of high conservation of the nucleocapsid gene: Forward: 5’-CAGTCAAATGGGCTGATGCA-3′; Reverse: 5’-AAAGGGCTATAAAGAGAATAAGGTATTCT-3′; Probe: 5’-CCCTGACGACCACGTTGTGGTTCA-3′, 5′ labeled with fluorescein amidite (FAM). Amplification and detection were performed using TaqMan Chemistry on the ABI 7500 instrument with the following conditions: 48°C for 30 min (1 cycle); 95°C for 10 min (1 cycle); and 95°C for 10 s followed by 60°C for 1 min (45 cycles). The amount of HCoV-229E in the supernatant (SN) was quantified per volume and expressed as percent relative to control. To determine intracellular HCoV-229E, relative quantification (RQ) was employed using RnaseP as the cellular normalizing gene: Forward: 5’-AGATTTGGACCTGCGAGCG-3′; Reverse: 5’-GAGCGGCTGTCTCCACAAGT- 3′; Probe: 5′- TTCTGACCTGAAGGCTCTGCGCG-3′, 5′ labeled with FAM.

### Immunofluorescence imaging of dsRNA

Cells were fixed with ice-cold absolute ethanol (Millipore, 100,983) for 15 min, washed, blocked for 30 min in 1% BSA (Sigma, A7906), and incubated with anti-double stranded RNA monoclonal antibody (SCICONS J2, 10,010,200) diluted 1:100 in PBS containing 1% BSA for at least 1 h at room temperature. Next, the cells were washed, incubated with IgG Alexa fluor 488-conjugated donkey anti mouse antibody diluted 1:800 in PBS containing 1% BSA and 1 μg/mL 4′,6-diamidino-2-phenylindole (DAPI) (Sigma, 32,670) for 40 min, washed, and mounted to slides. Images were collected using LSM 700 laser scanning confocal system (Zeiss Gottingen). Image analysis and quantification were done using the FIJI software.

### Plaque assay

MRC-5 cells were seeded in 12 well plates, 2 × 10^5^ cells/well. After 24 h, the medium was replaced with fresh 1 mL EMEM supplemented with 2% FBS, and the cells were infected with the supernatant from the 48-h long-term viral exposure experiments, 1.9 × 10^5^ virus copies per well and 5 serial 10-fold dilutions. At 2 hpi, the cells were washed to remove unbound virus and covered with EMEM supplemented with 2% FBS and 1.5% carboxymethylcellulose (Sigma, C4888). Four days later the cells were fixed with ice-cold absolute ethanol for 15 min and stained with 1% Cristal Violet (Mercury, 1,159,400,025) in 20% ethanol for 5 min at room temperature. Plaque forming units (PFU) were counted and divided by the dilution factor for obtaining the PFU per equal virus amount. To calculate the PFU for equal SN volumes the following equation was applied: number of plaques formed by identical virus dilutions/dilution factor x infection volume of SN in 1 mL infection media.

### Safety clinical study of TTFields application to COVID-19 patients

EF-37 study was a single arm, open label pilot study of NovoTTF-100 L (150 kHz TTFields) in hospitalized patients with COVID-19 disease, conducted between February 21 and March 15, 2021. The study was performed in the Hadassah medical center, Jerusalem, Israel. Ethics committee reviewed and approved the protocol. Trial registration number is NCT04953234. Written informed consent was obtained from all patients prior to any study related assessments/procedures being conducted.

Eligible for the study were hospitalized patients that were positive for COVID-19, and suffered from severe illness. Inclusion criteria were: age ≥ 18; hospitalized with diagnosis of COVID-19 infection per RT-PCR test within 72 h prior to treatment start; SpO_2_ ≤ 93% at sea level; lung involvement confirmed with chest imaging; able and willing to comply with all study procedures; and for female participants of childbearing age, use of highly effective contraception (a failure rate ≤ 1% per year when used consistently and correctly). Exclusion criteria included: receipt of any experimental treatment for COVID-19 prior to or during the study; assisted ventilation; critical illness, defined as respiratory failure (SpO_2_/FiO_2_ < 150), septic shock, and/or multiple organ dysfunction; significant comorbidities at baseline including clinically significant hematological, hepatic and renal dysfunction (defined as neutrophil count <1.5×10^9^/L and platelet count <100 × 10^9^/L, bilirubin >1.5 x ULN (Upper Limit of Normal), AST and/or ALT >2.5 x ULN, and serum creatinine >2.5 mg/dL), history of significant cardiovascular disease unless the disease is well controlled (second/third degree heart block, significant ischemic heart disease, poorly controlled hypertension, congestive heart failure, or symptoms of heart failure at rest), history of arrhythmia that is symptomatic or requires treatment, or history of any psychiatric condition that might impair patient’s ability to understand or comply with the requirements of the study or to provide consent; implantable electronic medical devices (e.g., pacemaker, defibrillator) in the upper torso; pregnancy or breast-feeding; known allergies to medical adhesives or hydrogel; or unwilling or unable to comply with the requirements of this protocol.

Overall, 10 patients were recruited. Enrollment was performed within 48 h of hospitalization, and patients were treated with TTFields delivered to the thorax while possibly receiving concomitant treatment with COVID-19 standard of care for a duration of 29 days or until no longer hospitalized with no limitations on activity (ordinal score of 1, *vide infra*). Clinical follow-up continued for 30 days post treatment end. The primary endpoint was the frequency and severity of treatment associated adverse events based on the Common Terminology Criteria for Adverse Events (CTCAE) v5.0. Secondary endpoints included: time to recovery, duration of hospitalization, all-cause mortality, incidence of intensive care unit (ICU) admission, non-invasive ventilation, high-flow oxygen use, invasive ventilation, and ECMO, clinical status on day 8, 15, 22, and 29 of treatment, and inflammatory status on day 3, 8, and 11 of treatment (until discharge).

Clinical status assessment was performed using an ordinal severity scale with 8 categories: (1) Not hospitalized, no limitations on activities; (2) Not hospitalized, limitation on activities and/or requiring home oxygen; (3) Hospitalized, not requiring supplemental oxygen–no longer requiring ongoing medical care; (4) Hospitalized, not requiring supplemental oxygen – requiring ongoing medical care (COVID-19 related or otherwise); (5) Hospitalized, requiring supplemental oxygen; (6) Hospitalized, on non-invasive ventilation or high flow oxygen devices; (7) Hospitalized, on invasive mechanical ventilation or ECMO; (8) Death. Time to recovery was measured from day of treatment start to the day the patient clinical status was defied as 1, 2, or 3 on the ordinary scale. For subjects discharged with a clinical status ≥4, the day of recovery was considered as the day of discharge. Subjects were censored as follows: lost to follow-up or terminated early prior to an observed recovery–at the day of their last observed assessment; completed follow-up but did not experience recovery–at their day 29 visit; and dead within day 29 (and prior to recovery)–at day 28.

TTFields were delivered to patients through four insulated surface arrays, placed on the patients’ skin surrounding the thorax to generate two perpendicular fields in the chest of the patient: A layer of adhesive hydrogel was applied to the arrays, the arrays were then applied to the thorax (that was shaved if needed as to obtain optimal coupling with the skin), and hypoallergenic medical tape was placed on top to secured the arrays. The arrays were replaced two to three times a week in order to maintain optimal coupling between the arrays and the patients’ skin. The arrays were attached to a field generator delivering currents of 1,414 mA in two sequential, perpendicular directions (1 s intermittently). The NovoTTF-100 L internal memory unit captured the time TTFields were delivered, thereby allowing objective report of device usage.

### Statistical analysis

*In vitro* experiments were presented as means ± SD. Statistical significance was calculated using GraphPad Prism 8 software (La Jolla). Differences were considered significant at values of: *p < 0.05, **p < 0.01, ***p < 0.001, and ****p < 0.0001. Clinical safety results were presented descriptively, and quantitative results were analyzed using the SAS 9.4 software. Time-to-event and incident endpoints were described with median and 95% confidence interval or interquartile range (IQR).

## Results

### TTFields inhibit viral entry

**Figure 1:**
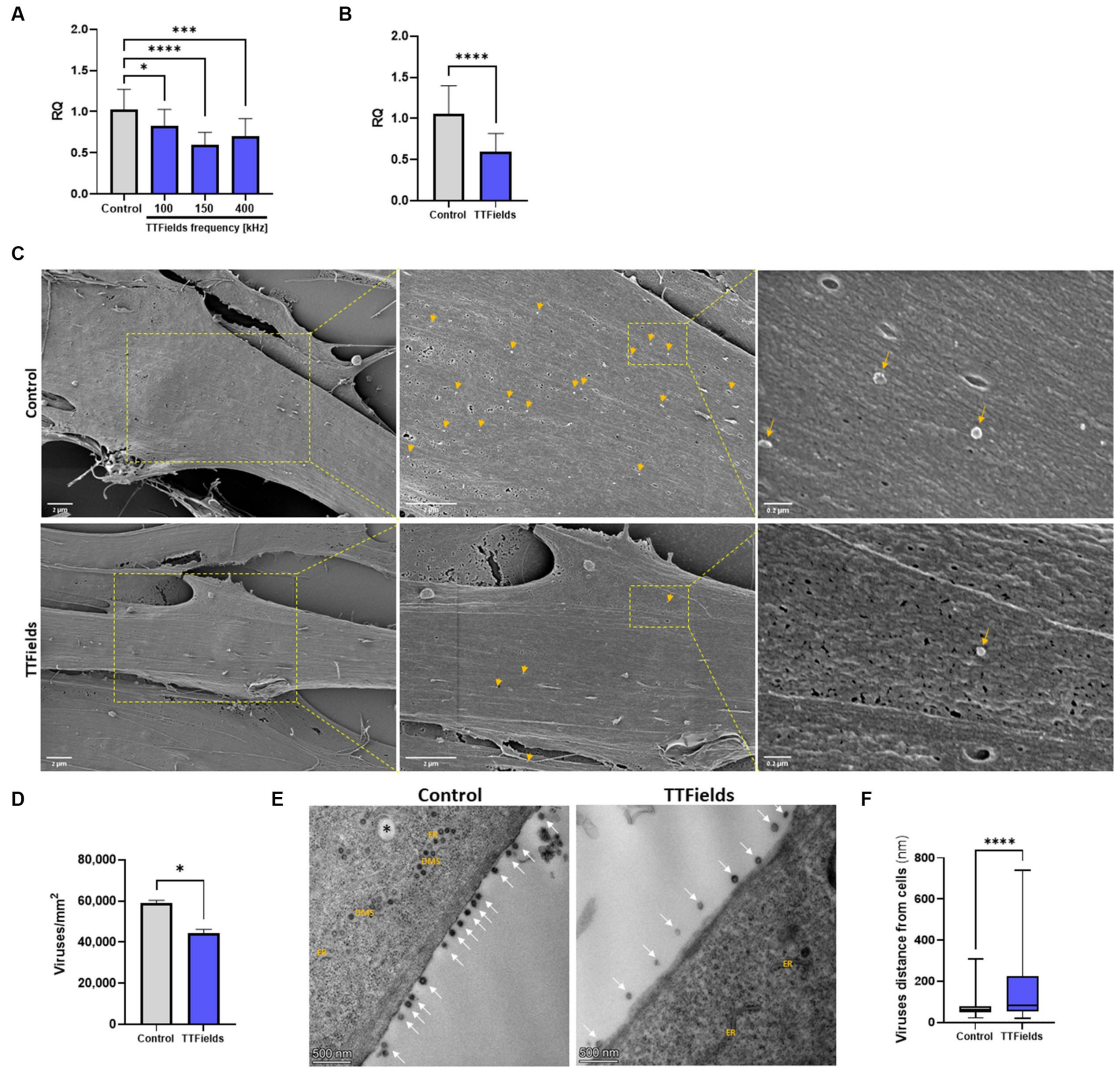
TTFields inhibit viral entry. (**A**) MRC-5 cells were infected with HCoV-229E (MOI = 0.01) while being exposed to TTFields at different frequencies, and cellular viral load measured by RT-qPCR at 2 hpi. (**B**) TTFields (150 kHz) were applied to MRC-5 cells while being infected with HCoV-229E (MOI = 0.01), followed by cellular viral load measured by RT-qPCR at 0.5 hpi. **(C,D)** TTFields (150 kHz) were applied to MRC-5 cells while being infected with HCoV-229E (MOI = 20), followed by SEM examination at 0.5 hpi to determine the number of viruses (designated with yellow arrows) attached to the cells. **(E,F)** MRC-5 cells were infected with HCoV-229E (MOI = 0.03) for 3 h, washed to remove extracellular virions, and then TTFields were applied. At 48 hpi, TEM examination was performed to determine the distance of the viruses (designated with white arrows) from the cells. MOI, multiplicity of infection; hpi, hours post infection; RQ, relative quantification; ER, endoplasmic reticulum; DMSs, double-membrane spherules; black asterisks, double-membrane vesicles (DMVs). Values are mean ± SD. *p < 0.05, ***p < 0.001, and ****p < 0.0001 relative to control; One-way ANOVA for A; Student’s *t*-test for **C**; Mann–Whitney test for **E**.

HCoV-229E is considered a valid, less infectious model for SARS-CoV-2 (Huang Y. et al., 2020), which we used for *in vitro* investigations to reduce biohazard risk. We first applied various TTFields frequencies to MRC-5 lung fibroblasts during 2 h of viral infection. RT-qPCR measurements revealed that TTFields produced significant 19, 42 and 32% reduction in cellular viral load relative to control (infected cells with no treatment) for 100, 150, and 400 kHz, respectively (Figure 1A). The most effective frequency (150 kHz) was selected for all subsequent experiments.

To examine TTFields effects on viral binding, infection time was shortened to 30 min, for which TTFields-induced reduction in cellular viral load was 43% (Figure 1B), similar to that seen for 2 h infections. Scanning and transmission electron microscopy (SEM and TEM, respectively) showed 25% reduction in the number of cell-bound virions for TTFields-treated relative to control cultures (Figures 1C,D); and that TTFields application significantly increased the distance of the virions from the cell membrane – 158 nm (range = 21–740 nm) compared to 75 nm (range = 21–307 nm) for control (Figures 1E,F). Overall, the results suggest that TTFields affect attachment of the virus to the cells.

### TTFields antiviral effect increases with longer treatment duration and reduces progeny infectivity

**Figure 2:**
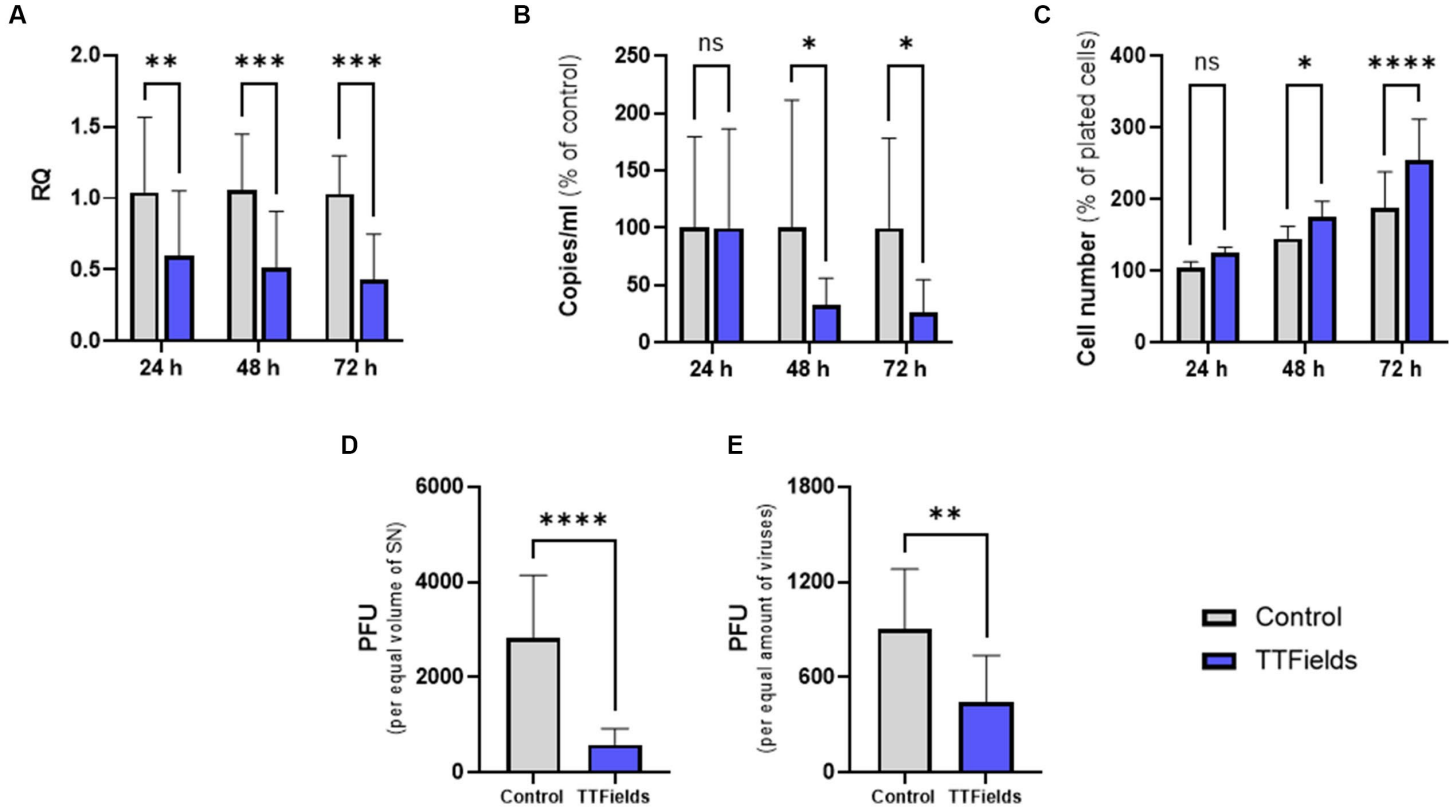
TTFields antiviral effect increases with longer treatment duration and reduces progeny infectivity. TTFields were applied for 24, 48 or 72 h to MRC-5 cells that were infected with HCoV-229E (MOI = 0.0001) during the first 3 h of treatment. The intracellular **(A)** and extracellular **(B)** viral amount was examined by RT-qPCR. Cell count was also measured **(C)**. Supernatants from 48 h exposures were added to MRC-5 cells (not exposed to TTFields at any stage), and plaque formation was determined for equal supernatant volumes **(D)** or virus amount **(E)**. MOI, multiplicity of infection; RQ, relative quantification; PFU, plaque forming units; SN, supernatant; ns, non-significant. Values are mean ± SD. *p < 0.05, **p < 0.01, ***p < 0.001, and ****p < 0.0001 relative to control; Sidak’s multiple comparison for **A**–**C**, Student’s *t*-test for **D** and **E**.

Cells infected with HCoV-229E while being exposed to TTFields for 24, 48 or 72 h post infection (hpi), showed a time dependent reduction of viral cellular load relative to control of 42, 51, and 58%, respectively (Figure 2A). Viral secretion, as determined by RT-qPCR analysis of the culture media, was very low at 24 hpi (Supplementary Figure S2), and not significantly affected by TTFields at this timepoint (Figure 2B). However, after 48 and 72 h of TTFields treatment, viral secretion decreased to 68 and 74% of control, respectively (Figure 2B).

Interestingly, following 24, 48, and 72 h application of TTFields to cells infected with the virus, cell counts increased by 19, 21, and 35% relative to control, respectively (Figure 2C). However, in the absence of virus cell growth levels were maintained following treatment with TTFields for 24 and 48 h and were slightly lower after 72 h (Supplementary Figure S3), indicating no direct effects of TTFields on cell proliferation. Altogether, these results suggest that TTFields were protecting the cells from the deleterious effects of the virus.

Supernatants from the 48-h viral exposures were subjected to plaque forming assays, without further application of TTFields. From equal supernatant volumes, plaque forming units (PFU) were 79% lower for virions formed under TTFields relative to control (Figure 2D). Surprisingly, 50% PFU reduction was seen per equal amounts of virions (Figure 2E), suggesting decrease not only in viral quantity but also in progeny infectious potential.

### TTFields inhibit viral replication

**Figure 3:**
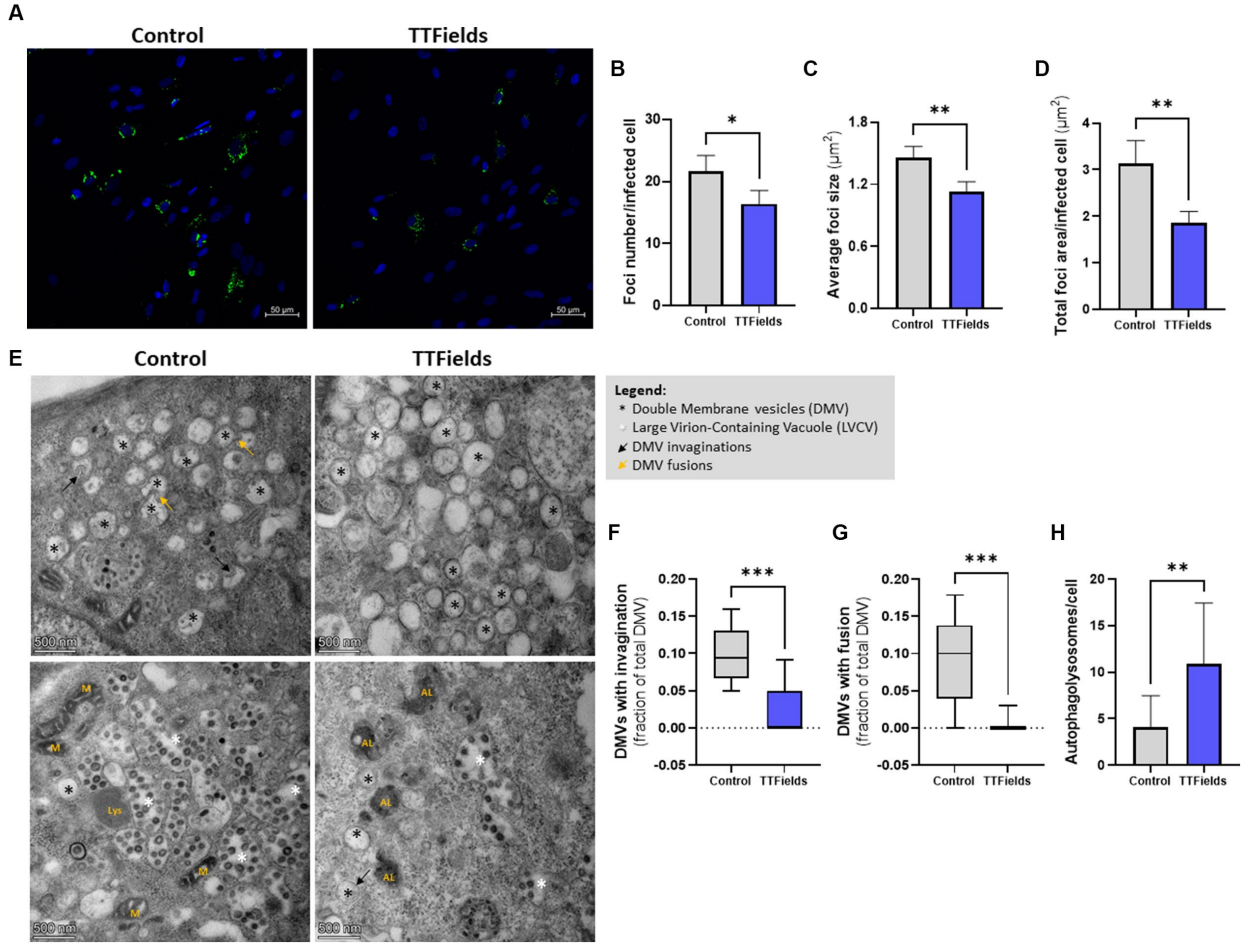
TTFields inhibit viral replication. **(A–D)** MRC-5 cells were infected with HCoV-229E (MOI = 0.01) for 3 h, washed to remove extracellular virions, and then TTFields were applied. At 24 hpi, dsRNA was detected by fluorescent microscopy with green staining, and cellular nuclei imaged with blue DAPI (x20 magnification). The number of foci per infected cell, average foci size, and total foci area per infected cell were quantified. **(E–H)** MRC-5 cells were infected with HCoV-229E (MOI = 0.03) for 3 h, washed to remove extracellular virions, and then TTFields were applied. At 48 hpi, TEM examination were performed to measure invaginations and fusion of DMVs, and to quantify autophagolysosomes (AL). MOI, multiplicity of infection; hpi, hours post infection; dsRNA, double stranded RNA; DMVs, double membrane vesicles; M, mitochondria; Lys, lysosome. Values are mean ± SD. *p < 0.05, **p < 0.01, ***p < 0.001, and ****p < 0.0001 relative to control; Student’s *t*-test for **B**–**D**, and **H**; Mann–Whitney test for **F** and **G**.

To explore effects on viral replication, cells were infected and washed to remove any extracellular virions before applying TTFields. These experiments were limited to 24 hpi, during which virion secretion is scarce (Supplementary Figure S2), and therefore minimal reinfection events are expected. RNA viruses utilize double membrane vesicles (DMVs) within the host cell as niches for RNA replication, a process involving the formation of double stranded RNA (dsRNA) (Wolff et al., 2020). To detect viral replication, the cellular presence of dsRNA was measured (Figure 3A). As the infection was performed identically, without TTFields, the number of infected cells was equal in control and treated cells (not shown). However, TTFields-treated cells contained 24% less dsRNA foci (Figure 3B), and those foci were 23% smaller in size (Figure 3C) and covered 41% less area per cell relative to dsRNA foci in control cells (Figure 3D). These results suggest the formation of fewer DMVs, and less viral RNA formation inside each DMV.

To verify this, DMV invaginations (Figure 3E black arrows, and Figure 3F) and homotypic fusions (Figure 3E yellow arrows, and Figure 3G), events needed for virion production, were quantified from TEM images; 70 and 93% lower event incidence, respectively, was demonstrated in cells exposed to TTFields relative to control. Overall, these results indicate reduced viral replication when TTFields were applied. Furthermore, a 3-fold increase in the number of autophagolysosomes (the result of autophagosomes fusion with lysosomes) were seen in infected cells treated with TTFields relative to control (Figure 3E marked with “AL”, and Figure 3H), indicating increased viral autophagic degradation.

### TTFields application together with remdesivir is beneficial *in vitro*

**Figure 4:**
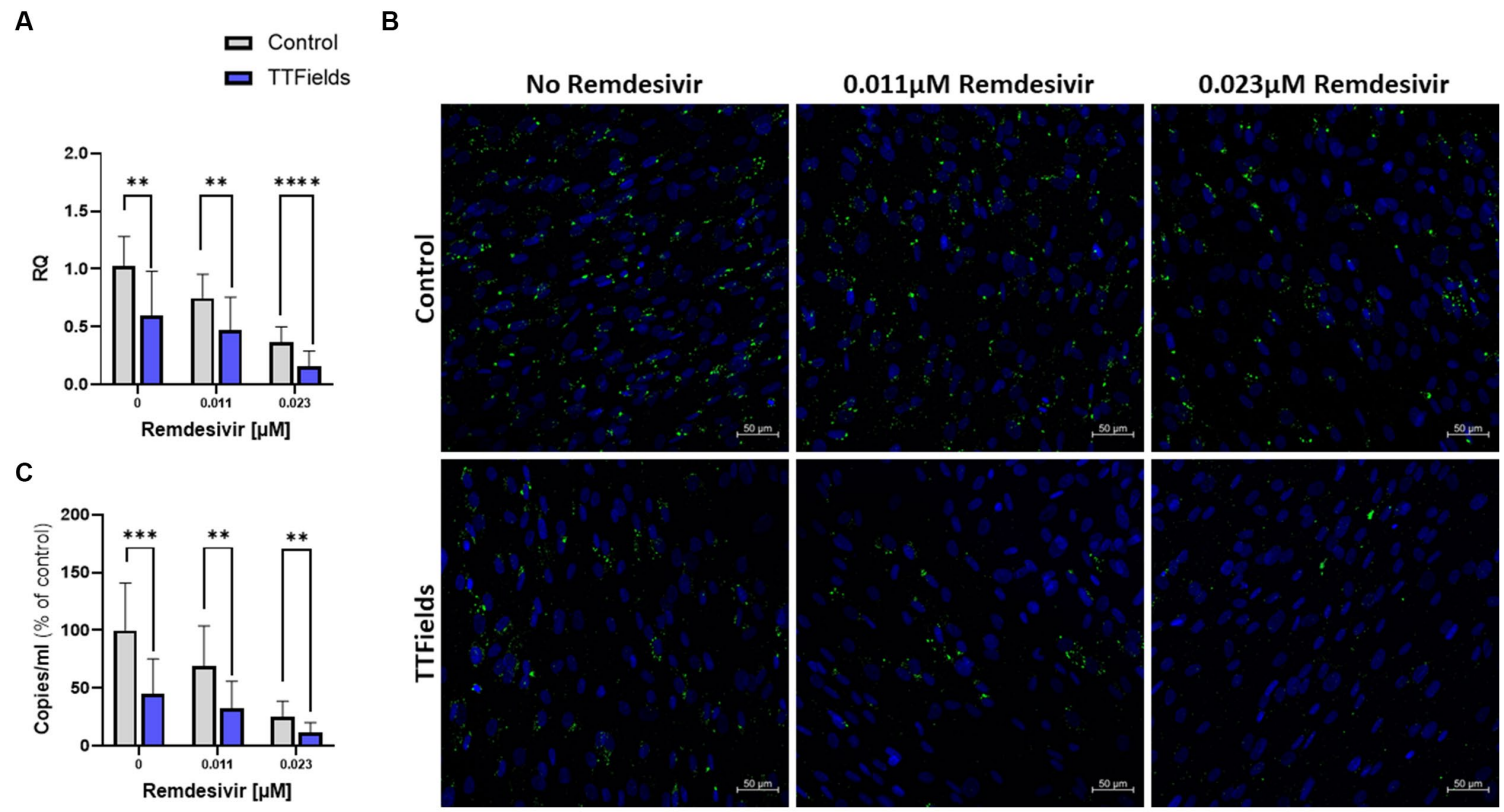
TTFields application together with remdesivir is beneficial *in vitro*. TTFields were applied for 48 h, alone or concomitantly with 0.011 or 0.023 μM remdesivir, to MRC-5 cells that were infected during the first 3 h of treatment with HCoV-229E (MOI = 0.01). The intracellular viral amount was examined by RT-qPCR **(A)** and by fluorescence detection of dsRNA with green staining, and cellular nuclei imaged with blue DAPI (x20 magnification) **(B)**, and the extracellular viral amount was examined by RT-qPCR **(C)**. MOI, multiplicity of infection; dsRNA, double stranded RNA; RQ, relative quantification. Values are mean ± SD. **p < 0.01, ***p < 0.001, and ****p < 0.0001 relative to control; Sidak’s multiple comparison.

Remdesivir, approved for hospitalized COVID-19 patients with severe disease, inhibits viral RNA replication (Parang et al., 2020; Pruijssers et al., 2020). Cellular viral load at 48 hpi was reduced by 27 and 65% for cells infected during treatment with 0.011 and 0.023 μM remdesivir, respectively, and by 42% for TTFields alone (Figure 4A). Concomitant application of TTFields with remdesivir reduced viral load by 54 and 85% for the low and high doses of remdesivir, respectively. The lower viral load following TTFields-remdesivir co-application was also evident from reduced levels of dsRNA within the cells relative to the monotherapies (Figure 4B). The number of virions secreted to the media was reduced, by 31 and 75% for 0.011 and 0.023 μM remdesivir alone, respectively, and by 55% for TTFields alone; while concomitant application resulted in a decrease of 68% for the low and 88% for the high doses of remdesivir (Figure 4C).

### Characteristics of patients enrolled in the pilot clinical study

Between February 21 and March 15, 2021, ten hospitalized COVID-19 patients were enrolled in the EF-37 study, designed to test the safety of 150 kHz TTFields therapy delivered to the thorax. Eligible patients were those with severe but not critical illness according to the World Health Organization (WHO) classification, i.e., demonstrating SpO_2_ < 94% at sea level with lung involvement, but without respiratory failure, septic shock, and/or multiple organ dysfunction. Patient population included both male and female patients, median age of 52 (range: 29–59), with most suffering from comorbidities (Table 1), representing well the population of COVID-19 hospitalized patients. All patients had an ordinal score for clinical improvement (OSCI) of 5 upon enrollment as per the study inclusion criteria. Patient inflammatory markers were elevated at baseline (Supplementary Table S1), though not above the threshold considered hyperinflammatory (Huang I. et al., 2020).

**Table 1:** Baseline characteristics and treatment details.

Characteristics	All patients (*N* = 10)	TTFields alone (*N* = 7)	TTFields with remdesivir (*N* = 3)	Historical control placebo (Beigel et al., 2020) (*N* = 521)	Historical control remdesivir (Beigel et al., 2020) (*N* = 541)
Age, years					
Mean (SD)	49.8 (10.5)	51.0 (8.6)	47.0 (16.2)	59.2 (15.4)	58.6 (14.6)
Median (Min - Max)	52.4 (28.7–59.4)	51.7 (39.7–59.4)	53.0 (28.7–59.3)	60 (21–95)	59 (21–94)
Sex, no. (%)					
Male	6 (60.0)	5 (71.4)	1 (33.3)	332 (63.7)	352 (65.1)
Female	4 (40.0)	2 (28.6)	2 (66.7)	189 (36.3)	189 (34.9)
Comorbidities, no./total no. (%)					
None	3/10 (30.0)	1/7 (14.3)	2/3 (66.7)	97/517 (18.8)	97/531 (18.3)
One Or More	7/10 (70.0)	6/7 (84.7)	1/3 (33.3)	420/517 (81.2)	434/531 (81.7)
Type of comorbidity, no./total no. (%)					
Hyperlipidemia	5/10 (50.0)	4/7 (57.1)	1/3 (33.3)	NA	NA
Ischemic heart disease	2/10 (20.0)	2/7 (28.6)	0/3	57/521 (11)	69/541 (13)
Obesity	3/10 (30.0)	3/7 (42.9)	0/3	234/518 (45.2)	242/531 (45.6)
Hypertension	4/10 (40.0)	4/7 (57.1)	0/3	264/519 (50.9)	269/532 (50.6)
Type 2 diabetes mellitus	4/10 (40.0)	3/7 (42.9)	1/3 (33.3)	158/519 (30.4)	164/532 (30.8)
Clinical status, baseline score of 5, no. (%)					
	10 (100)	7 (100)	3 (100)	203 (39.0)	232 (42.9)
Time from symptoms onset to study enrollment, days					
Median (IQR)	7 (3–11)	9 (3–15)	6 (3–7)	9 (7–13)	9 (6–12)
**Treatment with TTFields**					
Duration, days					
*N*	10	7	3	NA	NA
Median (IQR)	3.5 (2–4)	4 (2.5–4)	2 (1.5–3)	NA	NA
Usage, %					
*N*	10	6*	2*	NA	NA
Mean (SD)	55.9 (22.1)	67.2 (20.1)	44.8 (9.2)	NA	NA

*Excluding one patient (from each group) due to missing data.

Patients were treated with the NovoTTF-100 L device, continuously delivering 150 kHz TTFields therapy across their entire thorax (Giladi et al., 2014), via arrays attached to the skin (Supplementary Figure S1), together with standard-of-care treatment for COVID-19. As per the severity of their illness, all patients were treated with high flow oxygen, and nine of the ten patients were also subjected to steroid therapy. Three patients also required treatment with remdesivir.

Median treatment duration with TTFields was 3.5 days and mean daily device usage was 55.9% (Table 1). Treatment duration and usage were higher for patients treated with TTFields alone (4 days and 67.2%, respectively) relative to patients treated with concurrent remdesivir (2 days and 44.8%, respectively), due to two patients from the latter group who decided to discontinue early.

### TTFields demonstrated safety in COVID-19 patients

The clinical safety results are summarized in Table 2 and Supplementary Table S2. Two patients (20%) had the expected treatment-related pruritus (itchy skin, CTCAE grade 1). One patient (10%) fell, and one patient (10%) experienced back pain, both events occurring after TTFields therapy was stopped and were hence concluded not to be related to the treatment. One patient (10%) had severe (CTCAE grade 3–4) adverse events (AEs) during the study period. This patient withdrew consent from the study on day 2, and later experienced exacerbation of a previously known Pickwickian syndrome and multiple organ dysfunction syndrome. As a result, this patient required ICU admission and invasive ventilation and died 2 months after withdrawing consent. Since the patient had multiple comorbidities that are known risk factors for severe illness from COVID-19 (Gao et al., 2021) and was treated with TTFields for only 3 h, this outcome was concluded not to be related to TTFields. Overall, TTFields therapy did not demonstrate treatment-related safety concerns other than the expected skin toxicity.

**Table 2:** Adverse events by severity according to Common Terminology Criteria for Adverse Events (CTCAE) v5.0.

System organ class/preferred term	All patients (*N* = 10)	
	Low-medium (Grade 1–2)	Severe (Grade 3–4)
Number of patients with ≥ 1 AE	2 (20%)	1 (10%)
General disorders and administration site conditions		
Medical device site pruritus	2 (20%)	0
Multiple organ dysfunction syndrome	0	1 (10%)
Injury, poisoning and procedural complications		
Fall	1 (10%)	0
Musculoskeletal and connective tissue disorders		
Back Pain	1 (10%)	0
Respiratory, thoracic and mediastinal disorders		
Pickwickian syndrome	0	1 (10%)

### TTFields demonstrated preliminary effectiveness in COVID-19 patients

**Figure 5:**
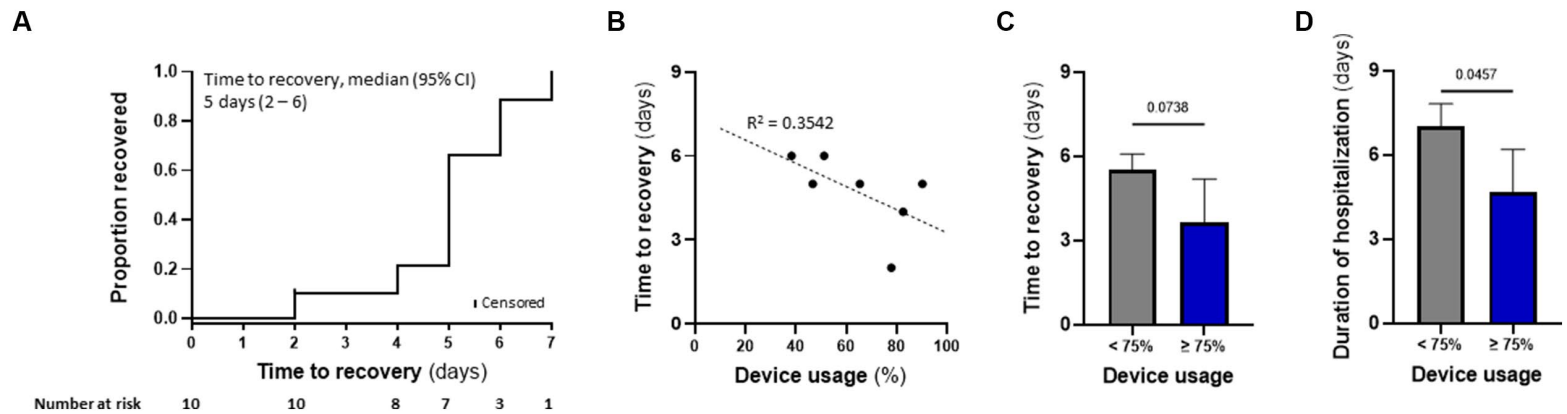
TTFields demonstrated preliminary effectiveness in COVID-19 patients, correlating with device usage time. **(A)** Kaplan–Meier estimates of cumulative recoveries in the intention-to-treat population. **(B)** Correlation between patient device usage and time to recovery in the per protocol population, excluding 2 patients with missing data. **(C)** Time to recovery and **(D)** duration of hospitalization for patients from panel b, divided to those with device usage of ≥75% (*N* = 3), and those with <75% device usage (*N* = 4). Values are mean ± SD. *p* value relative to control; Student *t*-test.

Patient’s median time to recovery was 5 days (Table 3; Figure 5A), about 30% shorter than reported for ACTT-1 trial (NCT04280705) COVID-19 patients treated with remdesivir alone (7 days), who had OSCI of 5 at study entry (Beigel et al., 2020). Median duration of hospitalization for the nine patients that completed TTFields treatment was 6 days (Table 3). During the study, none of the patients required non-invasive ventilation, high-flow oxygen, or ExtraCorporeal Membrane Oxygenation (ECMO) (Table 4).

**Table 3:** Effectiveness endpoints.

Secondary endpoints	All patients (*N* = 10)	TTFields alone (*N* = 7)	TTFields with remdesivir (*N* = 3)	Historical control placebo (Beigel et al., 2020) (*N* = 203)*	Historical control remdesivir (Beigel et al., 2020) (*N* = 232)*
Time to recovery, days					
*N*	10	7	3	203	232
Median (95% CI)	5 (2–6)	5 (2–not reached)	6 (5–not reached)	9 (7–10)	7 (6–8)
Duration of hospitalization, days					
*N*	9**	6**	3	NA	NA
Median (IQR)	6 (6–7)	6 (5–7)	7 (6–8)	NA	NA

*Including only results of patients with a baseline ordinal score for clinical improvement (OSCI) of 5. **Excluding one patient that withdrew consent after only 3 h of treatment.

**Table 4:** Additional effectiveness endpoints.

Additional secondary endpoints	All patients (*N* = 9)*
Incidence of all-cause mortality, no.	
Day 14	0
Day 28	0
Incidence of intensive care unit admission, no.	0
Incidence of non-invasive ventilation or high-flow oxygen use, no.	0
Incidence of invasive ventilation, no.	0
Incidence of Extra Corporeal Membrane Oxygenation use, no.	0

*Excluding one patient that withdrew consent after only 3 h of treatment and exacerbated after study end.

Median time to recovery (5 days) and duration of hospitalization (6 days) of the seven patients treated with TTFields alone were somewhat shorter than for the three patients treated with TTFields plus remdesivir (6 and 7 days, respectively). This median recovery time for the patients treated with TTFields alone was about 45% shorter than reported for the placebo-treated cohort from the ACTT-1 trial (9 days), who had OSCI of 5 at study entry (Beigel et al., 2020).

For the nine patients who completed the study, OSCI was 5, 2, and 1 for three (33.3%), five (55.7%), and one (11.1%) patients, respectively, on study day 8 (Table 5). On day 15, all nine patients had an OSCI of 1 or 2 (two (22.2%) and seven (77.8%) patients, respectively), a 31 and 41% increase compared to the ACTT-1 trial historical controls (with OSCI = 5 at study enrollment) on day 15 of treatment with remdesivir or placebo, respectively (Beigel et al., 2020). On day 22, all nine patients treated with TTFields had an OSCI of 1.

**Table 5:** Ordinal score for clinical improvement (OSCI) at specific time points, no. (%, [95% CI]).

Clinical status	All patients* (*N* = 9)**				Historical control placebo (Beigel et al., 2020) (*N* = 203)***	Historical control remdesivir (Beigel et al., 2020) (*N* = 232)***
	Day 8	Day 15	Day 22	Day 29	Day 15	Day 15
1	1 (11.1, [2–44])	2 (22.2, [6–55])	9 (100, [70–100])	9 (100, [70–100])	62 (30.5)	90 (38.8)
2	5 (55.6, [27–81])	7 (77.8, [45–94])	0	0	58 (28.6)	70 (30.2)
3	0	0	0	0	4 (2.0)	6 (2.6)
4	0	0	0	0	13 (6.4)	17 (7.3)
5	3 (33.3, [12–65])	0	0	0	18 (8.9)	25 (10.8)
6	0	0	0	0	7 (3.4)	5 (2.2)
7	0	0	0	0	21 (10.3)	13 (5.6)
8	0	0	0	0	20 (9.9)	6 (2.6)

*All enrolled patients had a baseline OSCI of 5, as per inclusion criteria. **Excluding one patient that withdrew consent after only 3 h of treatment. ***Including only results of patients with a baseline clinical ordinally score of 5.

Only five patients had C-reactive protein (CRP) levels measured on day 3 (four patients were already discharged and one withdrew consent), with 0.7 mg/dL reduction from baseline (Supplementary Table S1). On day 8, CRP levels were available for one patient (four more patients were discharged at this stage) and were 6.7 mg/dL lower than baseline and almost back to normal range. No other inflammatory markers were available after baseline.

### TTFields preliminary effectiveness in COVID-19 patients correlated with device usage time

Correlation between device usage and time to recovery was examined for seven patients, as one patient withdraw consent after only a few hours and two patients had missing data. Despite the small number of participants, there was a trend demonstrating that variance in recovery time could partially be explained by device usage (Figure 5B). Median time to recovery and duration of hospitalization were 4.0 and 5 days, respectively, for patients with usage ≥75% (*N* = 3) – the clinically recommended usage – compared to 5.5 and 7 days, respectively, for those with usage <75% (*N* = 4; Figures 5C,D). Overall, the high-usage group displayed almost 30% shorter time to recovery and duration of hospitalization relative to the low-usage group.

## Discussion

This is the first study to address the possible antiviral effects of TTFields. In this study the effects of TTFields on coronavirus infection were examined *in vitro*, using the HCoV-229E model strain and MRC-5 lung fibroblast.

**Figure 6:**
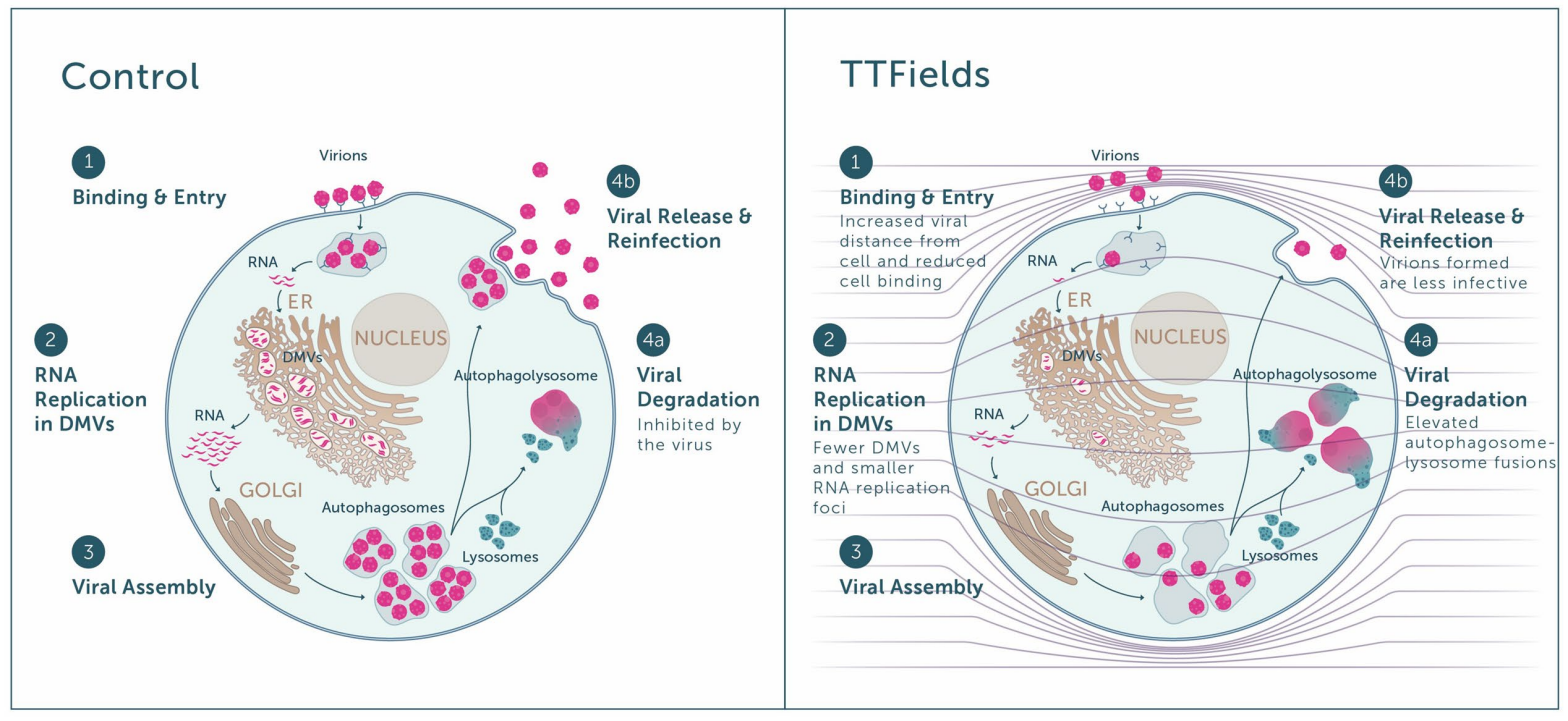
Overview of the antiviral effect of TTFields. Left panel: Virions bind to host receptors and enter the cell (step 1), in which viral RNA replication takes place inside double membrane vesicles (DMVs) (step 2), followed by viral assembly within the Golgi (step 3). The virus inhibits autophagosome-lysosome fusion and hence viral degradation (step 4a), enabling viral release and reinfection of new cells by progeny virions (step 4b). Right panel: When TTFields are applied to cells, the low cell membrane permittivity is expected to amplify field intensities in close proximity to the cells (see field lines across the scheme, and how they are condensed next to the cell membrane). Application of TTFields: interferes with the approach of virions to the cell, leading to lower receptor binding and cell entry (step 1); hinders viral replication, as indicative from the formation of fewer DMVs and smaller RNA replication foci therein (step 2); and induces autophagy, promoting viral degradation (step 4a) on the expense of release from the cell (step 4b). Virions formed under TTFields display lower infectivity.

Highest inhibition of viral cell entry was achieved with TTFields at a frequency of 150 kHz. The similar viral load seen when TTFields were applied during 30 min and 2 h of infection, together with the lower levels of viral-cellular attachments and higher virions-cell distance when TTFields were applied, suggest that TTFields interfere early in the viral entry process, during cellular attachment (Figure 6, step 1). Recently, simulation studies suggested that electric fields could affect the secondary and tertiary structure of the S protein of SARS-CoV-2 (Arbeitman et al., 2021). These alternations were suggested to inactivate or attenuate the virions. While in the current study TTFields were applied at lower intensities, the low-permittivity of the cell membrane is expected to amplify field intensities in close proximity to the cells (Wenger et al., 2015; Figure 6, right panel), and thus the field may reach sufficient intensity as to possibly affect S protein conformation.

As the virus life cycle includes secretion of progeny virions from the cells and repeated entry (Figure 6, step 4b), we also examined long-term effects of TTFields. These experiments demonstrated that TTFields effectively lowered viral intracellular load, accompanied by a decrease in virion secretion. While reduced release of virus particles could be a result of lower viral infection, additional possibilities are outlined hereafter, in accordance with other demonstrated effects of TTFields. Importantly, cell number was higher in infected cultures treated with TTFields relative to control, indicating that TTFields protected the cells from the harmful consequences of viral infection.

TTFields seem to impair the infectivity of progeny virions formed during treatment, as indicative from the lower PFU relative to control for identical virus amounts. This phenomenon may be due to the previously described conformational changes of the S protein induced by electric fields (Arbeitman et al., 2021). Alternatively, reduced progeny infectivity could be a downstream consequence of the previously demonstrated interference of TTFields with the dynamic process of microtubule assembly (Kirson et al., 2004; Giladi et al., 2015; Voloshin et al., 2020b), since microtubules support successful S protein incorporation into virions and are needed for release of infectious viral particles (Rudiger et al., 2016).

Cells that were treated with TTFields only during viral replication demonstrated decreases in the amount and size of dsRNA foci with lower levels of DMV remodeling events, indicative of a TTFields effect on viral replication (Figure 6, step 2). While the mechanism of this phenomenon is currently unclear and requires further investigation, it may contribute to the demonstrated observed reduction in viral secretion. Such interference in replication can potentially introduce defects to the viral RNA, rationalizing the lower infectivity of progeny virions formed under TTFields application.

Cells utilize autophagy to sense, control the growth of, and clear infecting viruses (Garcia-Perez et al., 2020; Gassen et al., 2021). However, the coronavirus hijacks the autophagy machinery, preventing autophagosome-lysosome fusion, thus protecting itself from degradation (Figure 6, step 4a; Garcia-Perez et al., 2020; Gassen et al., 2021). With TTFields there was evidence of increased autophagy, as demonstrated previously (Shteingauz et al., 2018; Voloshin et al., 2020a; Davidi et al., 2022), suggesting that the observed interference with viral secretion may be attributed in part to increased viral degradation.

Overall, the *in vitro* mechanistical studies showed that TTFields demonstrate antiviral activity, including inhibition of viral infection (Figure 6, step 1) and replication (Figure 6, step 2) and induction of viral degradation (Figure 6, step 4a), together leading to lower viral secretion (Figure 6, step 4b), and to formation of progeny virions with lower infectivity.

In the EF-37 pilot study, hospitalized COVID-19 patients suffering from a severe but not critical disease received TTFields therapy alone or concomitant with the broad-spectrum antiviral agent remdesivir (in both cases on top of oxygen and steroid treatment). Minimal toxicity of TTFields has been previously demonstrated in clinical studies with oncological patients, using the same electrical field parameters on the same anatomical site (Pless et al., 2013; Ceresoli et al., 2019; Vergote et al., 2019). In the current study, TTFields treatment was found to be safe, with no reported device-related serious AEs; and only 20% of patients experiencing mild skin irritation underneath the arrays. This rate of device-related skin reactions was lower than previously reported (60% for thoracic/abdominal indications, 35% for head indications; Vergote et al., 2019; Shi et al., 2020), possibly due to the shorter treatment duration of only a few days, compared to months of treatment for oncological patients.

The study was designed as a pilot study for examining safety, hence sample size was small, and all effectiveness outcomes should be treated as preliminary and exploratory. Albeit the very small sample size, TTFields showed signs of benefit for COVID-19 patients. Such indications include the shorter time to recovery and faster improvement in the clinical status of the patients who received TTFields (with or without remdesivir) relative to published data for treatment with placebo or remdesivir alone. Other indicators were hard to follow due to the rapid dynamics of the disease.

Furthermore, a correlation was suggested between device usage time and effectiveness outcomes, with shorter time to recovery and duration of hospitalization seen in the patients who used the device for ≥75% of the time. As the average 56% usage time in the current study was somewhat lower than that typically achieved in studies with oncological patients (65–75%) (Pless et al., 2013; Vergote et al., 2018; Rivera et al., 2019), and considering the preliminary results showing elevated effectiveness against COVID-19 with higher device usage, it may be hypothesized that increasing usage could provide additional clinical benefit in COVID-19 patients.

In this study, TTFields were applied to both tissue cultures and patients during the early stages of viral infection and over comparable durations. It may carefully be speculated that the antiviral effects observed for TTFields *in vitro* are also relevant in the clinical study, and that TTFields inhibited the potential rise of viral load in the COVID-19 patients. Maintaining low levels of viral load was shown to inhibit the possible decline of COVID-19 patients into the life-threatening ARDS stage (Blot et al., 2021; Choi et al., 2021; Magleby et al., 2021).

Deviations between clinical and preclinical results were seen in regard to the simultaneous TTFields and remdesivir application. Unlike the superiority demonstrated for the concomitant treatment in the preclinical setting, treatment of patients with TTFields plus remdesivir did not exhibit a benefit relative to TTFields alone. It should be noted that the patients prescribed remdesivir were those suffering from a more severe illness. Additionally, remdesivir is administered for a minimum of 5 days, which may account for the elongated treatment and hospitalization times of these patients. Furthermore, the patients treated with TTFields plus remdesivir had lower TTFields device usage and shorter treatment duration. Altogether, this may account for the lower clinical effectiveness for the concomitant treatment and the discrepancy from the preclinical results.

TTFields therapy delivery requires placement of arrays, connected to a field generator, on the patient’s thorax. This study demonstrates that patients are able to receive TTFields therapy for several days for more than 50% of the time, without interference with other medical interventions given to them during hospitalization. Since the device is portable, battery-operated, and intended for home use, TTFields can potentially be delivered to high-risk patients at their home, *a priori* reducing their probability of hospitalization. Since TTFields therapy is well tolerated, does not cause systemic toxicity, and is not expected to have contraindications with medications the patients may be receiving for managing their co-morbidities, it can be considered as a low-risk treatment option relative to agents that are associated with drug–drug interactions.

To conclude, the translational study described herein demonstrated a novel antiviral application of TTFields, an approved cancer treatment, as well as the preliminary safety and effectiveness of this therapy when applied to COVID-19 patients. Over time, coronavirus variants may arise with increased infectivity and possible resistance to the neutralizing antibodies that are elicited in convalescent and vaccinated individuals (Khateeb et al., 2021; Kimura et al., 2021), and to therapeutics that depend on the S protein structure. As TTFields are not tailored against any specific S protein amino acid sequence, but rather to the high protein polarity responsible for host receptor binding, they may be suitable for treatment of different COVID-19 variants, or possibly other types of viral infections, suggesting promise for this treatment option in the everchanging viral landscape.

## Data availability statement

The raw data supporting the conclusions of this article will be made available by the authors, without undue reservation.

## Ethics statement

The studies involving humans were approved by Hadassah medical center, Jerusalem, Israel ethics committee (trial registration: NCT04953234). The studies were conducted in accordance with the local legislation and institutional requirements. The participants provided their written informed consent to participate in this study. Ethical approval was not required for the studies on animals in accordance with the local legislation and institutional requirements because only commercially available established cell lines were used.

## Author contributions

AA: Conceptualization, Supervision, Writing – review & editing. HM: Conceptualization, Formal analysis, Investigation, Resources, Visualization, Writing – original draft. YaP: Conceptualization, Formal analysis, Investigation, Writing – original draft. NF: Conceptualization, Investigation, Writing – review & editing. NA: Conceptualization, Investigation, Writing – review & editing. SS: Writing – review & editing, Conceptualization, Resources. AN: Conceptualization, Resources, Writing – review & editing, Investigation. WM: Conceptualization, Investigation, Resources, Writing – review & editing. AK: Conceptualization, Investigation, Writing – review & editing. YK: Conceptualization, Investigation, Writing – review & editing. RS: Conceptualization, Writing – review & editing. DM: Conceptualization, Validation, Writing – original draft. OF: Conceptualization, Validation, Writing – original draft, Supervision. LB: Conceptualization, Formal analysis, Writing – review & editing. GL-S: Conceptualization, Formal analysis, Writing – review & editing. ED-O: Conceptualization, Writing – original draft. AH: Conceptualization, Writing – original draft, Formal analysis, Validation, Visualization, Writing – review & editing. MG: Conceptualization, Validation, Visualization, Writing – original draft, Writing – review & editing, Supervision. UW: Conceptualization, Resources, Supervision, Writing – review & editing. YoP: Conceptualization, Writing – review & editing. YM: Conceptualization, Resources, Supervision, Writing – review & editing. MM: Conceptualization, Resources, Supervision, Writing – review & editing.
