# Supplementary material for: Tumor Treating Fields (TTFields) demonstrate antiviral functions in vitro, and safety for application to COVID-19 patients in a pilot clinical study
Source: Front Microbiol. 2023 Nov 29;14:1296558. doi: 10.3389/fmicb.2023.1296558 (PMC10716356; doi:10.3389/fmicb.2023.1296558)
Supplement: Supplementary file 1 [file Table_1.pdf]

Supplementary Table S1: Inflammatory status at specific time points

Inflammatory status	All patients (N = 10)		
	CRP, mg/dL	D-dimer, mg/L	Ferritin, ng/mL
**Baseline**			
N	10	10	9
Mean (SD)	8.9 (8.2)	90.6 (284.7)	538.5 (493.1)
Median (Min-Max)	4.9 (0.9-26.2)	0.5 (0.2-901.0)	493 (7.1-1594)
**Day 3**			
N	5		
Mean (SD)	9.2 (5.8)		
Median (Min-Max)	10.0 (1.6-15.5)		
Change from baseline	-0.7		
**Day 8**			
N	1		
Mean (SD)	NA*		
Median (Min-Max)	NA*		
Change from baseline	-6.7		

* On day 8 CRP level reported only for one patient. Therefore, the mean and median are not reported.
