# Supplementary material for: Tumor Treating Fields (TTFields) demonstrate antiviral functions in vitro, and safety for application to COVID-19 patients in a pilot clinical study
Source: Front Microbiol. 2023 Nov 29;14:1296558. doi: 10.3389/fmicb.2023.1296558 (PMC10716356; doi:10.3389/fmicb.2023.1296558)
Supplement: Supplementary file 2 [file Table_2.pdf]

Supplementary Table S2: Serious adverse events (SAE)

System organ class / preferred term	All patients (N=10)
**Number of patients with ≥1 SAE**	
	1 (10%)
**General disorders and administration site conditions**	
Multiple organ dysfunction syndrome	1 (10%)
**Respiratory, thoracic, and mediastinal disorders**	
Pickwickian syndrome	1 (10%)
