# Supplementary material for: Tumor Treating Fields (TTFields) demonstrate antiviral functions in vitro, and safety for application to COVID-19 patients in a pilot clinical study
Source: Front Microbiol. 2023 Nov 29;14:1296558. doi: 10.3389/fmicb.2023.1296558 (PMC10716356; doi:10.3389/fmicb.2023.1296558)
Supplement: Supplementary file 3 [file Data_Sheet_1.pdf]

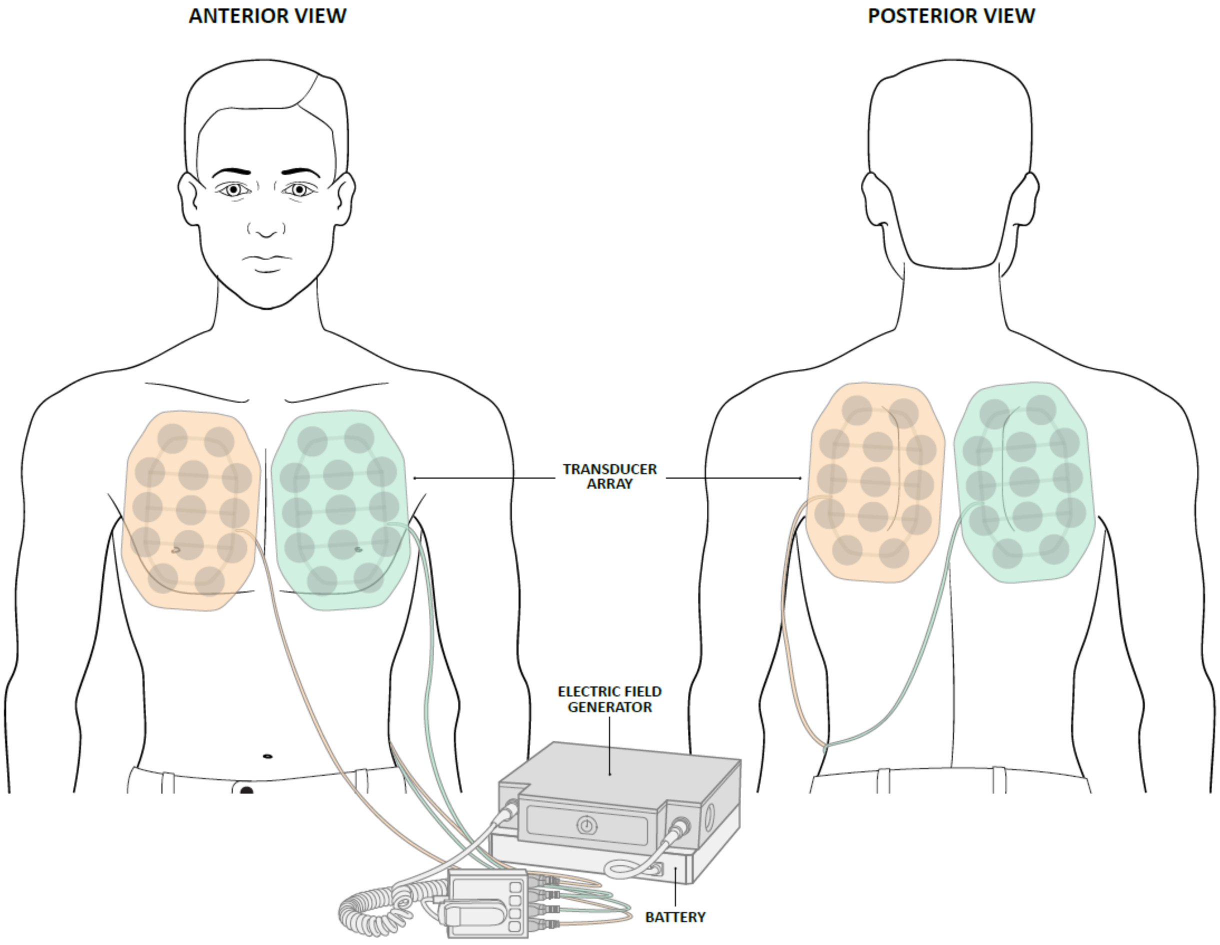

Supplementary Figure S1. Delivery of TTFields therapy to the patient thorax

TTFields are delivered via two pairs of transducer arrays positioned on the patient's thorax in a cross-chest layout (one pair on the right side of the chest and left side of the back, illustrated in yellow, and one pair on the left side of the chest and right side of the back, illustrated in green), that are connected to a battery-operated electric field generator.

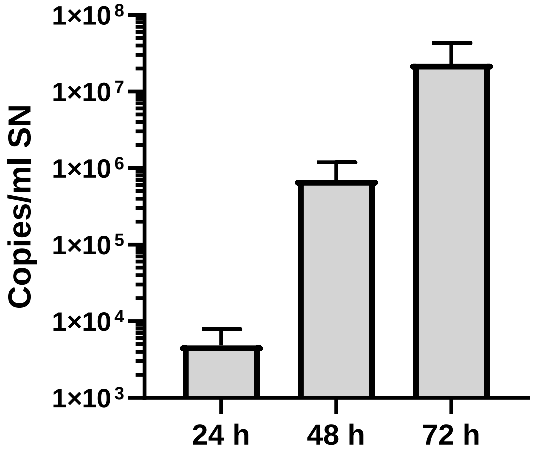

Supplementary Figure S2. Virus excretion levels over time

MRC-5 cells were infected with 0.01% HCoV-229E virus for 3 h, and the extracellular viral amount was examined by RT-qPCR at 24, 48, and 72 hpi. SN = supernatant.

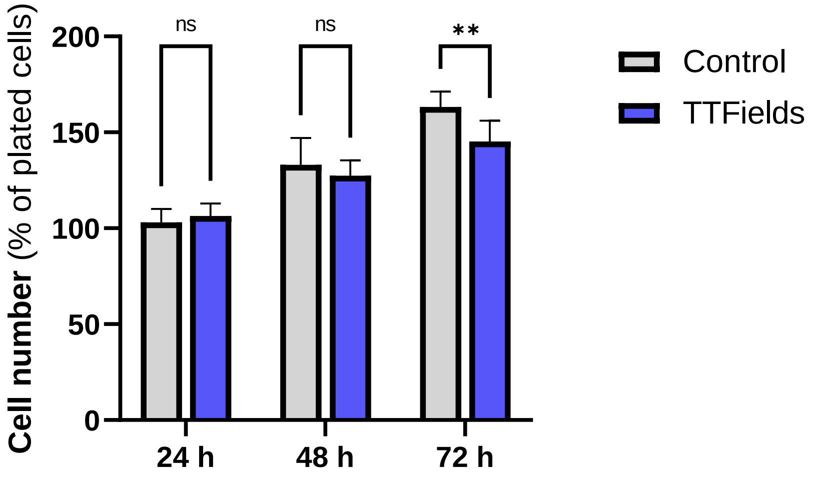

Supplementary Figure S3. Effect of TTFields on MRC-5 cells with no virus

MRC-5 cells were exposed to TTFields for 24, 48 or 72 h, and cell count was measured. ns = non-significant. Values are mean ± SD. ***p* < 0.01 relative to control; Sidak's multiple comparison.
